# Host development overwhelms environmental dispersal in governing the ecological succession of zebrafish gut microbiota

**DOI:** 10.1038/s41522-020-00176-2

**Published:** 2021-01-19

**Authors:** Fanshu Xiao, Wengen Zhu, Yuhe Yu, Zhili He, Bo Wu, Cheng Wang, Longfei Shu, Xinghao Li, Huaqun Yin, Jianjun Wang, Philippe Juneau, Xiafei Zheng, Yongjie Wu, Juan Li, Xiaojuan Chen, Dongwei Hou, Zhijian Huang, Jianguo He, Guohuan Xu, Liwei Xie, Jie Huang, Qingyun Yan

**Affiliations:** 1 Environmental Microbiomics Research Center, School of Environmental Science and Engineering, Southern Marine Science and Engineering Guangdong Laboratory (Zhuhai), Sun Yat-sen University, 510006 Guangzhou, China; 2 Key Laboratory of Aquatic Biodiversity and Conservation of Chinese Academy of Sciences, Institute of Hydrobiology, Chinese Academy of Sciences, 430072 Wuhan, China; 3 College of Agronomy, Hunan Agricultural University, 410128 Changsha, China; 4 Key Laboratory of Biometallurgy of Ministry of Education, School of Minerals Processing and Bioengineering, Central South University, 410083 Changsha, China; 5 State Key Laboratory of Lake Science and Environment, Nanjing Institute of Geography and Limnology, Chinese Academy of Sciences, 210008 Nanjing, China; 6 Department of Biological Science, GRIL, TOXEN, Ecotoxicology of Aquatic Microorganisms Laboratory, Université du Québec à Montréal, Succursale Centre-Ville, Montréal, QC Canada; 7 Key Laboratory of Ecological Impacts of Hydraulic-Projects and Restoration of Aquatic Ecosystem of Ministry of Water Resources, Institute of Hydroecology, Ministry of Water Resources and Chinese Academy of Sciences, 430079 Wuhan, China; 8 State Key Laboratory of Applied Microbiology Southern China, Guangdong Institute of Microbiology, Guangdong Academy of Sciences, 510070 Guangzhou, China

**Keywords:** Microbial ecology, Microbial genetics

## Abstract

Clarifying mechanisms underlying the ecological succession of gut microbiota is a central theme of gut ecology. Under experimental manipulations of zebrafish hatching and rearing environments, we test our core hypothesis that the host development will overwhelm environmental dispersal in governing fish gut microbial community succession due to host genetics, immunology, and gut nutrient niches. We find that zebrafish developmental stage substantially explains the gut microbial community succession, whereas the environmental effects do not significantly affect the gut microbiota succession from larvae to adult fish. The gut microbiotas of zebrafish are clearly separated according to fish developmental stages, and the degree of homogeneous selection governing gut microbiota succession is increasing with host development. This study advances our mechanistic understanding of the gut microbiota assembly and succession by integrating the host and environmental effects, which also provides new insights into the gut ecology of other aquatic animals.

## Introduction

Ecological succession has been a central theme in ecology for more than 120 years^1^. However, the mechanism underlying gut microbiota succession in aquatic animals remains elusive^2^, especially at a relatively long-term scale^3^. Understanding host-associated microbial succession needs to consider interactions with host genetics and ecology^4,5^, which undoubtedly increases the complexity to elucidate mechanisms governing the gut microbial community succession^6^. Although many studies indicated that both ecological and evolutionary forces could affect the succession of intestinal microbiota in terrestrial animals^7,8^, much less is known about that in aquatic fish gut ecosystem, which also colonizes with diverse microbial communities^9–11^. Fish encompass nearly one half of the vertebrate diversity^12^ and are considered as the most successful vertebrates evolved on Earth^13^. Their successful evolution may not be possible without the help of gut microbiota^9,10^. Of course, fish in turn provide gut microorganisms with appropriate habitats and necessary nutrients, and protect the gut microbiota from adverse disturbances (e.g., pathogen invasion)^14^. Such host-microbiota interactions are especially important for fish health, immunity, metabolism, development, reproduction, behavior, as well as defense of disease^9,10,15^.

The mechanism governing aquatic fish gut microbiomes may be utterly different from those of terrestrial mammals^14^. As oviparous fish cannot get heritable microorganisms from their mother as that of viviparous mammals delivered vaginally^16^. Recent studies found that fish gut microbiota was closely correlated with host genetics^17^, immunology^18^, physiology^19^, and ecology^4,20^, suggesting that such host-associated features should be important in governing fish gut microbiota. However, some other studies indicated that environmental factors were crucial forces shaping the fish microbiome^21,22^. Theoretically, all microorganisms colonize in the fish gut ecosystem are expected to be derived from the surrounding environment. However, fish are also able to retain some low abundant microorganisms in the environment, meaning that the bacteria (even the poor colonizer) obtained from the environment could further evolve to be more prolific colonizers^23^. Moreover, bacteria increasingly better adapt to colonization of fish by repeatedly moving from host to host through the external water environment^24^. So, it would be better to consider gut and water microbiota as a metacommunity to get a full understanding of fish-associated microbiomes. However, the contribution of fish microbiota to the ecosystem diversity of metacommunity (γ_Ecosystem_) is rarely addressed^25^. But in fact, any community is governed both by processes that occur within the local community and all linked communities^26,27^.

So far, the mechanism underlying fish-microbiota-environment associations remains controversial^13^. Host selection due to fish genetics could be a primary deterministic process to colonize core gut microbiota in zebrafish, regardless of whether they were recently collected from natural habitats or reared for generations in different labs^28^. Moreover, the relative importance of non-neutral processes (e.g., microbe-microbe interactions, active dispersal, selection) governing zebrafish gut microbiota was found to be increased as host development^29^. Some other studies fund that ecological processes governing gut microbiota are variable across fish development^16,30^. So far, only a few fish microbiome studies considered the host developmental issue, but the fish microbial diversity and composition could be significantly affected by the fish development^16,29–33^. Also, some other uncontrollable field factors (e.g., geographic distances, habitats, diets) may overestimate the discrepancy of fish gut microbiome among different studies. Fortunately, laboratory zebrafish allow us to dissect resultant effects from the environment and host under controllable conditions, and have been a powerful model for dissecting host-microbial interactions^15^. In addition, the succession of gut microbiota across zebrafish life cycle can be examined within a short period (i.e., three to four months), which greatly shortens the experimental time and therefore can reduce unexpected biases from long-term experiments. Another advantage of using zebrafish model is we can perform whole gut ecosystem analysis due to its small size, so that the overall microbial diversity can be precisely estimated.

This study aimed to clarify how host development and environment dispersal affect the assembly and succession of fish gut microbiota using the manipulated zebrafish model. We hypothesized that the host would have much stronger effects on the fish gut microbiota succession than the environment due to host genetics, immunology, and gut nutrient niches. So, we first tested whether fish hatched in different environments could assemble similar microbiota. Second, we examined whether established gut microbiota could be disturbed after zebrafish husbandry environment was switched. Third, we quantified the relative effects of host development and environmental dispersal on the gut microbiota succession. We found that the gut microbiota assembly and succession were mainly governed by host development rather than environmental dispersal. This study not only clarifies the effects of the host development, hatching environment, and environmental transition on the fish gut microbiota succession, but also provides new insights into our mechanistic understanding of gut ecology of other aquatic animals.

## Results

### Different water environments constructed for manipulation of zebrafish

**Fig. 1:**
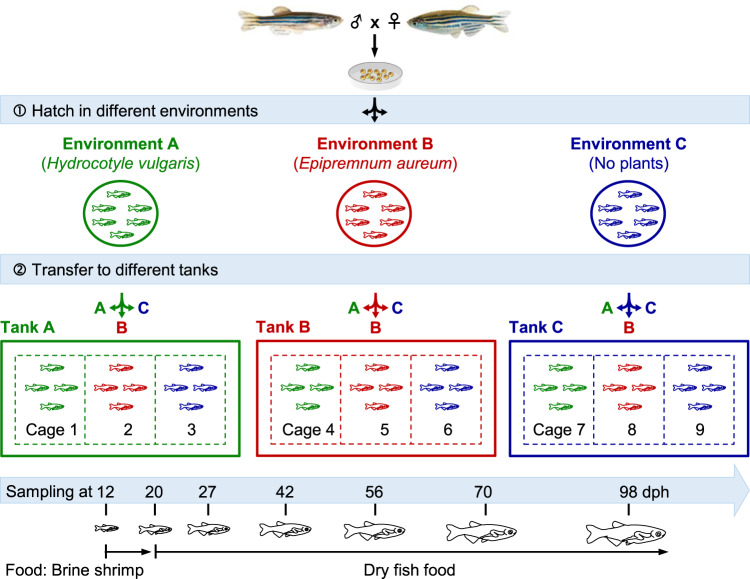
Experimental setup for testing host and environmental effects on the ecological succession of gut microbiota across zebrafish development. Zebrafish were manipulated under three different environments (A, B, and C). First, zebrafish embryos belong to a single sibship were hatched in three independent circular plates with water from environments A, B, and C, respectively. Second, zebrafish hatched from different environments (indicated by green, red, and blue, respectively) were transferred from plates to tanks at 12 days post-hatching (dph) and raised in small net cages (dotted box). Gut samples were collected from different cages across zebrafish development from 12 to 98 dph. The colored lines, fish and letters in green, red, and blue corresponding to environments A, B, and C, respectively. Cages 1, 5, 9 represented zebrafish kept in original environments, and the other cages represented zebrafish subjected to switched environments.

**Fig. 2:**
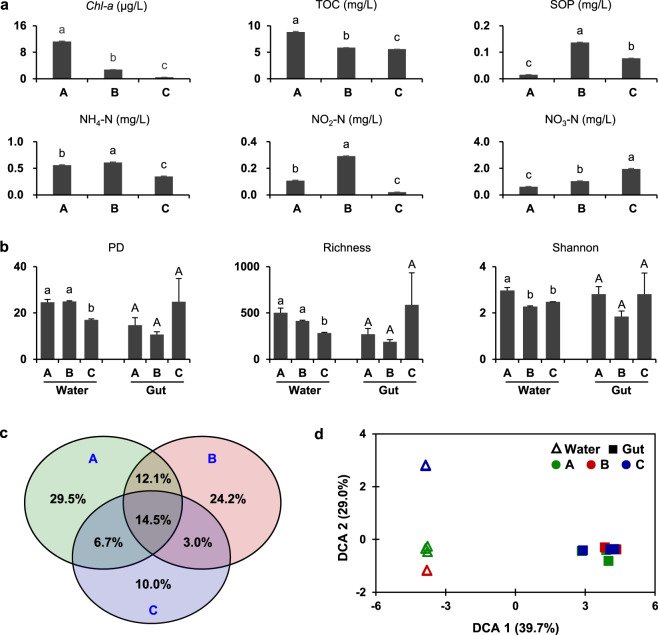
Chemical and microbial characteristics of the three constructed water environments and zebrafish gut microbiota colonized at 12 dph. **a** Chemical factors of water. Mean values were plotted with standard errors, and the variation among environments were tested through an ANOVA with least-significant-difference (LSD) tests. The presence of different letters denoted significant differences among environments, and the same letter indicated no significant difference. *Chl-a* Chlorophyll *a*, SOP soluble orthophosphate, TOC total organic carbon. **b** alpha-diversity of water and gut microbiotas. PD phylogenetic diversity. **c** Venn diagram of water microbiotas. The Venn diagram represent proportions of shared OTUs (operational taxonomic units) across environments over the total number of OTUs detected in all environments, but it does not provide quantitative data on the OTUs. **d** detrended correspondence analysis showing the dissimilarity of water and gut microbiotas at 12 dph (days post-hatching).

To examine the effects of surrounding environments on the initial colonization and subsequent succession of gut microbiota, we constructed three different water environments to manipulate zebrafish (Fig. 1). We found that all investigated water chemical factors were significantly different among environments A, B, and C (*p* < 0.05). Specifically, the environment A showed the highest concentrations of *Chl-a* and total organic carbon (TOC), whereas the soluble orthophosphate (SOP), NH_4_-N and NO_2_-N in the environment B were the highest, and the environment C had the highest NO_3_-N (Fig. 2a). The microbial richness and phylogenetic diversity (PD) of water microbiotas in environments A and B were significantly higher than those of environment C (*p* < 0.05), whereas the Shannon diversity in the environment A was statistically higher than those of B and C (*p* < 0.05, Fig. 2b). Also, the Venn diagram indicated that the composition of microbial OTUs in the three environments were considerably different: only 3.0–14.5% of the detected OTUs were shared by two or three environments, whereas 63.7% were unique OTUs (Fig. 2c). The three water environments were also clearly separated by the abundance-weighted DCA ordination (Fig. 2d). Thus, it is expected that the constructed environments would provide distinct microbial species pools for colonizing fish gut microbiota.

### Zebrafish gut microbiota assembly and turnover in original environments

**Fig. 3:**
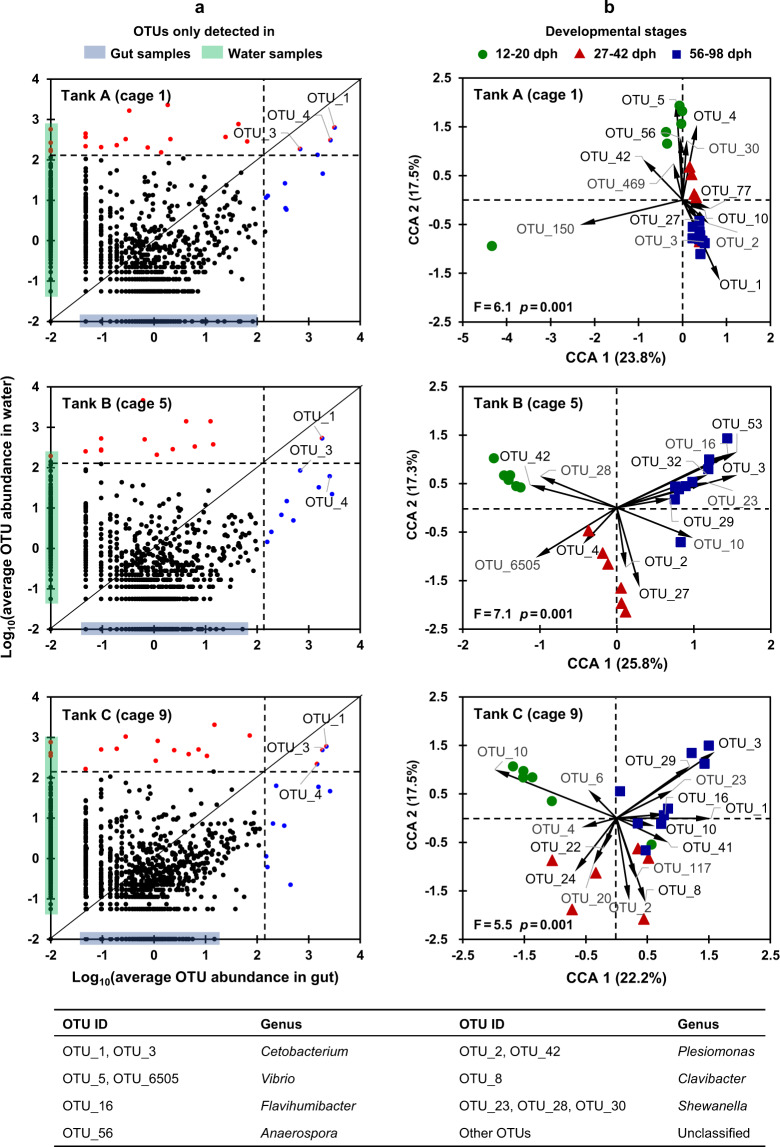
The average abundance for each detected OTU and the variation of gut microbial communities explained by the top 20 OTUs. **a** the average abundance for each OTU across water and gut microbiotas. The microbial OTUs that were equally abundant in gut and water samples fall along the diagonal line, whereas those enriched in the water or gut samples fall above or below the line, respectively. Dashed lines marked 1% of the average abundance in water or gut samples, respectively. The OTUs averagely >1% in gut or water samples are shown in blue and red, respectively. The red and blue mix points indicate OTUs dominated in both gut and water microbiotas, and the black points indicate OTUs averagely <1% in gut or water samples. **b** canonical correspondence analysis (CCA) showing gut microbiota variation explained by the top 20 OTUs. Each point represents a gut microbial community of individual zebrafish, and arrows represent the contribution of the top 20 bacterial OTUs.

To test whether different water environments would affect the initial assembly of fish gut microbiota, we analyzed water and gut microbial communities as well as their relationships. First, we found that the same batch of zebrafish embryos hatched in different environments assembled similar gut microbial communities at 12 dph, which cannot be separated in the DCA ordination based on Bray–Curtis distances (Fig. 2d). The alpha-diversity in terms of PD, richness and Shannon (Fig. 2b) also showed no significant differences among zebrafish hatched in environments A, B, and C (*p* > 0.05). Then, we further explored whether water environments would influence the turnover of the established gut microbiota in zebrafish that consistently kept in original environments (i.e., cages 1, 5, 9). The results showed that gut microbiotas were also different from water microbiotas across zebrafish development (Supplementary Fig. 1). Specifically, only three OTUs (OTU_1: *Cetobacterium*, OTU_3: *Cetobacterium*, and OTU_4: *Aeromonadaceae*, their detailed taxonomy please see Supplementary Table 1) on average >1% in both gut and water samples; most of other relatively prevalent OTUs detected in the gut samples were not abundant in water samples (Fig. 3a). Based on the Bray–Curtis distance analysis, gut microbiotas of zebrafish kept in each original environment were clearly separated according to fish developmental stages (i.e., 12–20, 27–42, and 56–98 dph, Supplementary Fig. 1). The top 20 OTUs of gut microbiota, which explained 39.7–43.1% of community variations, also showed clear distinctions among fish developmental stages (Fig. 3b).

In each original environment (i.e., cages 1, 5, 9), the dominant OTUs in zebrafish gut also showed similar succession patterns. Specifically, zebrafish at 12–20 dph colonized relatively high abundances of OTU_5 (*Vibrio*) and OTU_4 (*Aeromonadaceae*), which cumulatively accounted for >50.0% of the microbial abundance (Supplementary Fig. 2a). At 27–42 dph, OTU_2 (*Plesiomonas*) became dominant (16.2–26.7%), whereas OTU_5 (*Vibrio*) decreased to only 5.0–5.8% of the total abundance. However, OTU_4 (*Aeromonadaceae*) was generally kept at a relatively high abundance (10.6–31.4%) from 12 to 42 dph (Supplementary Fig. 2a). At 56–98 dph, two *Cetobacterium* members (OTU_1 and OTU_3) increased considerably and accounted for 40.0% of the total abundance, but OTU_4 (*Aeromonadaceae*) decreased to 8.2–11.0% (Supplementary Fig. 2a). However, the OTUs with relatively high abundances in water environments (e.g., *Flavobacterium* members of OTU_7 and OTU_11, Supplementary Fig. 2b), which theoretically have high opportunity to enter the gut through environmental dispersal, did not become dominant taxa in gut ecosystems. By contrast, the low abundant microorganisms in the water became prolific colonizers in zebrafish gut but depended on the host development. Thus, both gut microbial community composition and the abundance of dominant OTUs in zebrafish gut all showed clear developmental stage-specific patterns.

### Zebrafish gut microbiota succession after environmental transitions

**Fig. 4:**
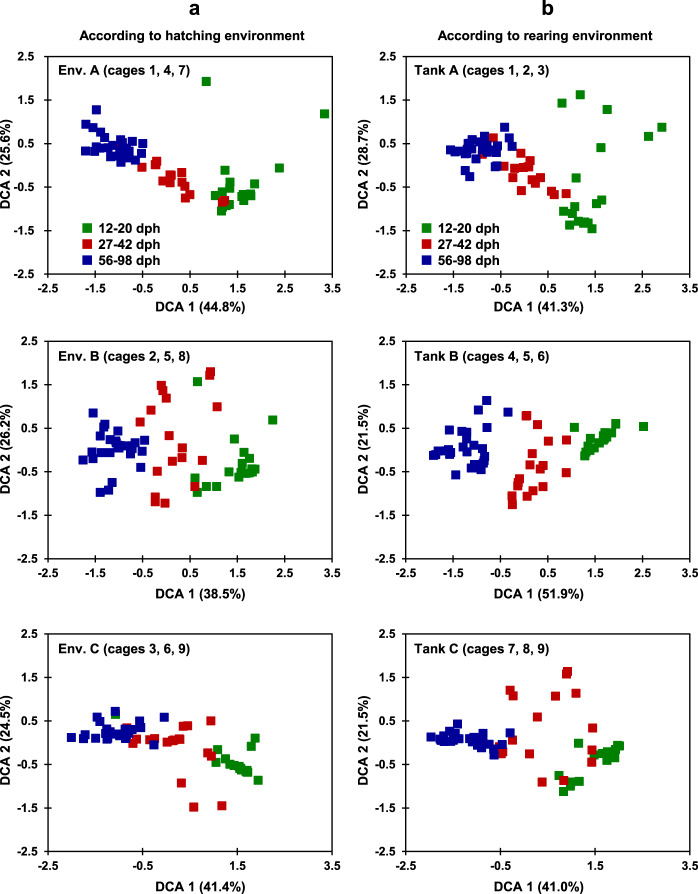
Detrended correspondence analysis (DCA) showing environmental effects on the succession of gut microbiota across zebrafish development. **a** According to hatching environment. **b** According to rearing environment. Zebrafish hatched from different environments (Env.) or raised in different environments showed similar gut microbial succession patterns across host development.

After 12 dph, some zebrafish were subjected to pairwise environment transitions to further test the influence of environmental dispersal on the gut microbiota succession. We tried to answer two questions: (i) whether zebrafish hatched in the same environment but raised in different environments tend to show different succession patterns? (ii) do zebrafish hatched from different environments but raised in the same environment tend to have similar succession patterns? Our results indicated that the ecological succession of gut microbial communities was mainly associated with zebrafish developmental stages regardless of their hatching environment (Fig. 4a) or rearing environment history (Fig. 4b). Gut microbial communities of zebrafish within each tank could be clearly split into three groups corresponding to three developmental stages (i.e., 12–20, 27–42, and 56–98 dph, Fig. 4). It could be further confirmed by dissimilarity tests (PERMANOVA, *p* < 0.05, Table 1). However, analyses performed between zebrafish in original environments (cages 1, 5, 9) and switched environments (cages 2, 3, 4, 6, 7, 8) within any stage were not significantly different (*p* > 0.05, Supplementary Fig. 3 and Supplementary Table 2). Similar stage-specific patterns were observed when we analyzed zebrafish across all tanks to test the influence of overall environmental dispersal on gut microbiota succession (Table 1 and Supplementary Fig. 4a), but had no environment-specific patterns (Supplementary Table 2 and Supplementary Fig. 4b). Moreover, the gut microbial communities at any stage were also significantly different from those of corresponding water microbial communities (*p* < 0.05, Supplementary Table 3). The succession patterns of zebrafish gut microbiota after environmental transitions was similar to those kept in original environments, which also split seven timepoints into three stages. Thus, the hatching environments and environmental transitions did not significantly affect the zebrafish gut microbiota succession, which appeared to be mainly determined by the host development.

**Table 1:** Permutational multivariate analysis of variance (PERMANOVA) showing the community differences of gut microbiota among zebrafish developmental stages.

	Bray–Curtis		Jaccard	
	*F*	*p*	*F*	*p*
*Hatched in environment A but raised in different tanks*				
12–20 dph vs 27–42 dph	12.32	0.001	3.83	0.001
12–20 dph vs 56–98 dph	37.54	0.001	5.09	0.001
27–42 dph vs 56–98 dph	18.09	0.001	4.11	0.001
*Hatched in environment B but raised in different tanks*				
12–20 dph vs 27–42 dph	10.04	0.001	3.51	0.001
12–20 dph vs 56–98 dph	35.32	0.001	5.82	0.001
27–42 dph vs 56–98 dph	17.72	0.001	3.41	0.001
*Hatched in environment C but raised in different tanks*				
12–20 dph vs 27–42 dph	11.29	0.001	3.00	0.001
12–20 dph vs 56–98 dph	36.43	0.001	4.42	0.001
27–42 dph vs 56–98 dph	13.30	0.001	3.16	0.001
*Hatched from different environments but raised in tank A*				
12–20 dph vs 27–42 dph	9.32	0.001	3.92	0.001
12–20 dph vs 56–98 dph	29.80	0.001	5.48	0.001
27–42 dph vs 56–98 dph	14.54	0.001	4.98	0.001
*Hatched from different environments but raised in tank B*				
12–20 dph vs 27–42 dph	29.18	0.001	5.67	0.001
12–20 dph vs 56–98 dph	61.07	0.001	10.86	0.001
27–42 dph vs 56–98 dph	30.39	0.001	7.35	0.001
*Hatched from different environments but raised in tank C*				
12–20 dph vs 27–42 dph	11.04	0.001	3.41	0.001
12–20 dph vs 56–98 dph	37.61	0.001	6.27	0.001
27–42 dph vs 56–98 dph	18.18	0.001	3.88	0.001
*Across all tanks*				
12–20 dph vs 27–42 dph	32.84	0.001	8.36	0.001
12–20 dph vs 56–98 dph	108.91	0.001	13.72	0.001
27–42 dph vs 56–98 dph	47.75	0.001	9.12	0.001

*dph* day post-hatching.

### Host development determined the ecological succession of zebrafish gut microbiota

**Fig. 5:**
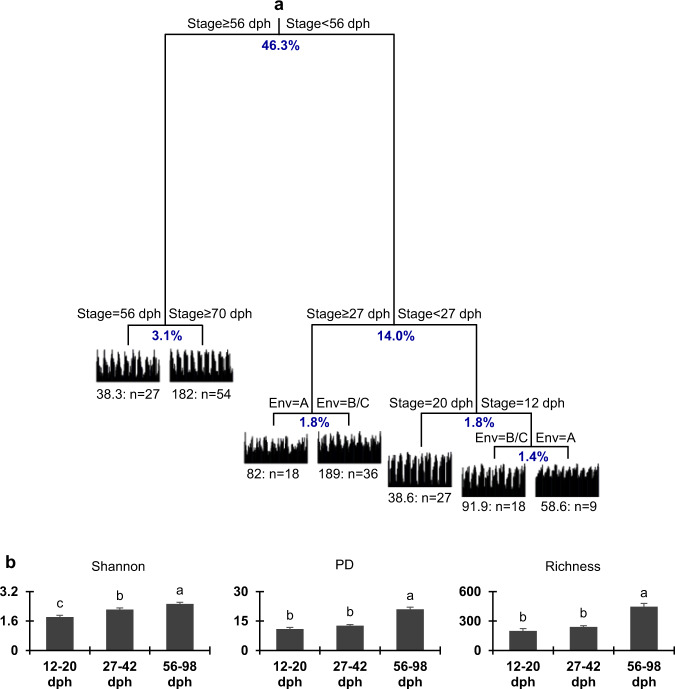
Zebrafish gut microbial diversity mainly associated with the developmental stage. **a** the multivariate regression tree (MRT) analysis performed base on the Bray-Curtis distance with interactions of different factors (i.e., developmental stage, environment, transition and food). **b** alpha-diversity of Shannon, phylogenetic diversity (PD), and richness were plotted with standard errors. The variations among environments were tested through an ANOVA with least-significant-difference (LSD) tests. The presence of different letters denotes significant differences among environments, and the same letter indicates no significant difference.

As both hatching environments and environment transitions did not significantly affect the assembly and succession of zebrafish gut microbiota, we subsequently focused on the qualitative and quantitative contributions of different factors (i.e., developmental stage, environment, transition, and food) to the gut microbiota succession. The multivariate regression tree (MRT) analysis explained 68.3% of the microbial diversity variance (Fig. 5a). Specifically, the diversity estimates of gut microbiota were first split by developmental stage (46.2%), which was the most important factor influencing the diversity and succession of gut microbiota, while the water environment only explained a small proportion of variance at early stages (1.4% at 12 dph, 1.8% at 27–42 dph). The alpha-diversity in terms of Shannon, PD and richness (Fig. 5b) also showed significant differences among different developmental stages (*p* < 0.05). The general linear model (GLM) analysis indicated that the developmental stage was the only significant predictor for shaping the alpha-diversity (*p* < 0.05, Supplementary Table 4). Specifically, the variance of PD, richness or Shannon was mainly explained by the developmental stage (*r*^2^ = 33.4–45.9%, *p* < 0.001); the environment only explained 3.4% of the richness significantly (*p* < 0.05), but the transition and food showed no significant explanation (*p* > 0.05). In addition, hierarchical partitioning variance of these alpha-diversity indices showed that the contribution of developmental stage (64.3–82.4%) was much stronger than those of environment (1.2–9.8%), transition (5.8–14.8%), or food (9.7–11.1%; Supplementary Table 5). Mantel tests performed using both Bray–Curtis and Jaccard distances indicated that the developmental stage was the strongest factor that significantly underlying the beta-diversity (*r* > 0.5, *p* < 0.001), and partial Mantel tests estimate the correlations between diversity matrix and stage whilst controlling for the effects of environment/transition/food were kept consistent (*r* > 0.5, *p* < 0.001, Table 2). However, the transition had no significant correlation with the beta-diversity (*p* > 0.05); and the environment and food only showed a weak (|*r*| ≤ 0.2) correlation to the beta-diversity (Supplementary Table 6). Therefore, zebrafish developmental stage was the major predictor of gut microbiota succession, overwhelming those of environment, transition and food. However, we should acknowledge that the impact of maternal/chorion variation as a source of gut microbiota variation was not explored here.

**Table 2:** Summary statistics for Mantel and partial Mantel tests of correlation between gut microbiota (*M*) and developmental stage (*S*).

Test type	Test statistic	Bray–Curtis		Jaccard	
		*r*	*p*	*r*	*p*
Mantel	*r*(*MS*)	0.66	<0.001	0.52	<0.001
Partial Mantel	*r*(*MS.E*)	0.65	<0.001	0.52	<0.001
	*r*(*MS.T*)	0.65	<0.001	0.51	<0.001
	*r*(*MS.F*)	0.62	<0.001	0.51	<0.001

The Mantel statistic *r*(*AB*) estimates the correlation between two matrices *A* and *B*. Whereas, the partial Mantel *r*(*AB*.*C*) statistic estimates the correlation between *A* and *B* whilst controlling for the effects of *C*. The *E, T*, and *F* indicate environment, transition, and food, respectively.

### Ecological processes governing zebrafish gut microbiota succession

**Fig. 6:**
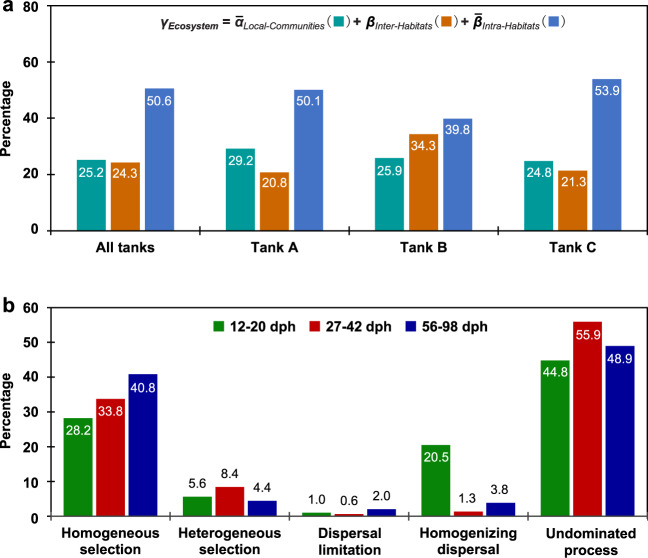
The quantification of metacommunity diversity and ecological processes. **a** hierarchical partitioning of the metacommunity diversity at multiscale. The ecosystem diversity (*γ*_Ecosystem_) of each tank or across all tanks was partitioned into contributions of *α*_Local-Communities_ (mean diversity of each water or gut sample), *β*_Intra-Habitats_ (mean of diversity within water or gut habitats), and *β*_Inter-Habitats_ (sum of diversity between water and gut habitats). **b** the quantified major ecological processes governing the gut microbial communities. The percentages (numbers on the individual bars) are given the relative contribution of each known process to the community succession at different stages, and the remaining parts attributed to undominated process.

Ecological process analyses were performed to explore mechanisms governing the ecological succession of gut microbiota across zebrafish development. We found that the ecosystem diversity (γ_Ecosystem_) across all tanks or within each tank showed similar contributions from different parts of diversity. Specifically, the metacommunity was mainly contributed by the mean of diversity within water or gut habitats (*β*_intra-__Habitats_, 39.8–53.9%, Fig. 6a), indicating gut microbiota is also an important contributor to the ecosystem microbial diversity. By contrast, the mean diversity of each water or gut sample (*α*_Local-Communities_) and the sum of diversity between water and gut habitats (*β*_inter-Habitats_) represented 24.8–29.2% and 20.8–34.3% (Fig. 6a) of the overall diversity, respectively. The null model test also indicated that the observed community similarities within zebrafish developmental stages were significantly differed from those of randomly permutated communities (*p* < 0.05), showing relatively high deterministic ratios (Supplementary Table 7). The quantified ecological processes confirmed that the succession of gut microbiotas in zebrafish was governed by strong deterministic process (Fig. 6b). Specifically, the homogeneous selection, which causes community composition to be similar under consistent environmental conditions, was responsible for 28.2–40.8% of gut microbial variation and it increased with host development. The heterogeneous selection, which causes community composition to be dissimilar under different environmental conditions contributed an additional 4.4–8.4% of variation. By contrast, the homogenizing dispersal at the early stage was also important (20.5%), but the contribution of dispersal limitation was much weak (only 0.6–2.0%) throughout the fish development (Fig. 6b). Thus, the zebrafish gut microbiota succession appeared to be mainly governed by stage-dependent selection, and the homogenizing dispersal was only important at the early stage.

## Discussion

Understanding the ecological succession of fish gut microbiota and underlying mechanisms facilitates the host metabolism, health and environmental adaptation^10,24^, and therefore has become a central theme of gut ecology. This study clarified the major forces governing the assembly and succession of fish gut microbiota across host development under different water environments. We found that the gut microbiota was mainly determined by the host development rather than hatching environments or environmental transitions. Thus, the results generally supported our hypothesis that the host development would overwhelm environmental dispersal in governing the gut microbial community succession across fish development due to host genetics, immunology, and gut nutrient niches.

The host genetics is known as the most important force to provide a primary selection of gut microbiota^24,34^. With a short period (12 days) of monitoring *Poecilia sphenops*, the fish gut microbiota found to be mainly driven by deterministic host effects independent of water microbiota^35^. Many other studies also showed that gut microbial communities in different fish species were considerably different due to the host genetics or phylogeny^17,36,37^, and the same fish species could colonize core gut microbiota despite radical differences in host provenance and domestication status^28^. Also, host sub-genomes (with hybrid fish lineages)^38^ and host genotypes (among-population differences)^39^ could affect microbial community in the fish gut ecosystem. However, those studies were always focused on a particular short developmental stage of fish. Interestingly, recent studies suggested that fish development could be one of the most important factors driving the succession of gut microbiota. For example, Burn et al.^29^ found that the relative importance of non-neutral processes for gut microbial community assembly in zebrafish increased over host development. This study agreed with such conclusion due to the deterministic process of homogenous selection increased with fish development for the assembly of gut microbiota. These studies along with some others^32,33^ started a new era to understand fish gut microbiota as the diversity and composition of microbial communities were found to be variable over zebrafish development. Our previous studies also indicated that aquaculture fish (e.g., *Ctenopharyngodon idellus, Siniperca chuatsi* and *Silurus meridionalis*) could assemble a respective gut microbial community ~3 dph^16^, followed by stage-specific patterns across fish development^16,30,31^, which is consistent with the microbial community turnover across host development in other animals such as shrimp^40^, insect^41,42^, bird^43^, mouse^44^, and human^45,46^. Thus, valid comparisons across studies about the gut microbiota should at least strive to use consistent ages of the sampled hosts^32^.

This study aimed to further understand the assembly and succession of gut microbiota by integrating effects of host development, hatching environment, and environmental transition. We found that the effects of zebrafish development on the gut microbiome were much stronger than those from the environment. The observed stage-specific gut microbiota succession patterns could be attributed to three aspects. First, the colonization of intestinal microbes in zebrafish initially occurs when zebrafish mouth opens at approximately day 3 post-fertilization^47^. This is coincident with the lumen formation of intestinal tract, and the whole gastrointestinal tube is opened at day 6 post-fertilization^47^. By this time, the yolk sac is almost consumed^47^, and the larvae start to get outside foods and simultaneously colonize diverse microbial species^48^. The colonized microbes in turn can further facilitate intestinal development in zebrafish^49^. That means the functions assisted by the colonized microbes in gut ecosystem may vary across zebrafish development, especially during the period of developing organs, and therefore showed stage-specific gut microbiota. Second, host immunity is also known as an important force to affect the fish gut microbiota. Although zebrafish innate immunity at early stages (e.g., before 18 dph) only has a small effect on the diversity of gut microbiota^15^, their adaptive immunity increases the role of selective processes in gut microbiota assembly^18^. Other animals with high immune functions could support much smaller microbial populations in the intestine than those with immune-compromised hosts^50^. In this study, the gut microbiota succession in zebrafish showed a clear split between 12–20 and 27–42 dph. This could associate with the development of zebrafish adaptive immune system, which is only functional after 25 dph^51^. Third, host-associated microbes could also benefit from their host with a protected and nutrient-rich niche^33,52^, which may considerably differ within a host at different developmental stages. Thus, the assembly of gut microbiota suggested to be largely driven by the nutrient landscape created by host diet and secretions^53^. The availability of nutrients in gut ecosystems, as well as gut motility and digestive processes varied across zebrafish development may also contribute to the stage-specific gut microbiota.

We found that the homogeneous selection was the major deterministic process governing gut microbiota, but the impact of maternal/chorion variation as a source of gut microbiota variation was not explored here. Moreover, the homogeneous selection increased with zebrafish development, resulting in similar microbial communities as gut environments within a stage tend to be similar. The homogenizing dispersal was only important at the early stage, as the newly formed gut ecosystem initially have no pioneer species to exclusive dispersal of other microbial species. This is consistent with previous study, which suggested that the relative importance of neutral process decreased over host development^29^. It also explained why microbial communities in larval zebrafish were more similar to environmental communities than those of adult fish^32^. Besides the selective pressures from host, the absence of significant relationships between gut microbiota and water microbiota may also attribute to different conditions in gut and water habitats, which could cause community composition to be dissimilar due to the process of heterogeneous selection. Generally, only the microbial species in the surrounding water environment with the tolerance to conditions in the gut ecosystem could colonize and thrive as part of the gut microbiota. Of course, we also should acknowledge that some other processes such as microbe-microbe interactions and active dispersal currently classified as undominated process may also contribute to the succession of fish gut microbiota.

Compared with strong host effects on the succession of gut microbiota across zebrafish development, the environmental influences derived from hatching environment or environmental transition were much smaller. This is consistent with previous findings in zebrafish^29,32^ and those in grass carp^16^, showing that gut microbial communities were different from the surrounding environment. Some other aquatic animals also exhibited considerably different gut microbial communities from those in the surrounding water^31,40,54,55^ or sediments^56^. Rudi et al.^57^ identified stable core gut microbiota in Atlantic salmon during the freshwater-to-saltwater transition. Besides the selective pressures from the host as discussed above, the interhost microbial transmission through the water is also a significant force that help fish shaping similar gut microbiota in different individuals^15,24^. In this study, we found that the overall diversity of metacommunity was mainly attributed to the mean diversity within gut or water habitats, indicating gut microbiotas contribute significantly to the metacommunity of the rearing ecosystem. Generally, the host-associated microbiota is strongly shaped by the factors that influence microbial survival and persistence in the gut ecosystem. The host gut habitat is a key issue for selecting the species able to colonize and thrive as part of its microbiota^58,59^. The successful colonizers in the gut ecosystem should have selective advantages of traits to occupy space, obtain resources, and avoid removal via excretions^23,24^. Their success of colonization in fish gut with relatively high abundances in turn can affect the metacommunity of the rearing system due to ceaseless communication (i.e., feeding and defecation) with surrounding water.

In summary, this study sought to resolve the effects of host development, hatching environment and environmental transition on the gut microbiota succession from larvae to adult fish. Although the impact of maternal/chorion variation as a source of gut microbiota variation was not explored, we found that zebrafish developmental stage substantially explained the ecological succession of gut microbial communities and dominant OTUs. Moreover, the ecological succession of zebrafish gut microbiota was mainly governed by stage-dependent homogeneous selection due to host effects, and the environmental impacts of hatching environments and environmental transitions were much smaller. These findings expand our current understanding of the ecological succession of gut microbiota across fish development, and also provide new insights into gut ecology of other aquatic animals.

## Methods

### Experimental setup and zebrafish (*Danio rerio*) husbandry

We investigated the gut microbiota assembly and succession in wild-type zebrafish (AB strain) from larvae to adults. We first established three different water environments using independent glass tanks (130 × 30 × 40 cm). Specifically, environments A and B were planted with *Hydrocotyle vulgaris* and *Epipremnum aureum*, respectively, and their roots were kept in water to exchange materials between plants and water, which were distinct from environment C with no plants. After plants grew for 30 days, water samples were collected from the left, center, and right of each tank as three replicates to evaluate the environmental differences among A, B, and C. The water microbiota and water chemical factors (i.e., NH_4_–N, NO_3_–N, NO_2_–N, Chlorophyll *a*, soluble orthophosphate, and total organic carbon) were monitored as described previously^60^.

The three different water environments constructed were then used to manipulate zebrafish and test our hypothesis in three aspects. First, to test possible effects of different water environments on the assembly and turnover of gut microbiota, the same batch of fertilized zebrafish embryos were randomly assigned to three independent circular plates (~1000 embryos per plate) and hatched with waters from environments A, B, and C, respectively (Fig. 1). Second, to test the effect of environmental transitions on the established gut microbiota, 100 individuals of zebrafish hatched from each environment were transferred to each of the three independent tanks (one tank corresponding to an environment, Fig. 1) at 12 days post-hatching (dph), but raised in small net cages fixed in the tanks. In each tank, there were three cages to separate fish transferred from environments A, B, and C, but the cages within a tank were connected and the microbial community in the water of each tank represented a metacommunity. This design could help us to explore whether zebrafish hatched from different environments tend to have similar succession patterns. Zebrafish raised in each cage had equivalent density to ensure our comparisons and interpretations of the effects derived from environmental transitions are reliable. Third, to explore the relative importance of host and environmental effects on the gut microbiota succession, fish and water samples were collected with 1–4 weeks interval (i.e., 12, 20, 27, 42, 56, 70, and 98 dph, Fig. 1). All zebrafish used herein belong to a single sibship and theoretically should have similar effects from host genetics, but the environmental dispersal was distinct due to different hatching environments and subsequent environmental transitions.

Zebrafish were raised under standard laboratory conditions according to the method described previously^30^. In brief, a stable water temperature (28 ± 0.5 °C) as well as 14/10-h light/dark cycle was controlled. No additional food was provided before the yolk sac was completely consumed (4 dph). Then, zebrafish were fed with cultured *Paramecium* (5–8 dph), 20 µm mesh filtrated boiled egg yolk (9–11 dph), live brine shrimp (12–19 dph), and a standard dry fish food from 20 dph onward (Fig. 1).

### Sampling procedures and microbial community DNA extraction

Three fish were randomly sampled per tank per treatment per timepoint as replicates, and each fish was used as an individual specimen in subsequent experiment. The intestines of larval individuals were immediately removed aseptically under a dissecting microscope as described previously^30^, and the juvenile/adult individuals were aseptically removed their intestines directly. The whole intestine of each fish was kept in a sterile 1.5-mL tube as a single sample, ensuring that the intestinal microbial diversity in each gut ecosystem is fully estimated. All protocols involved in the fish experiments were approved by the Institutional Animal Care and Use Committee of the Institute of Hydrobiology, Chinese Academy of Sciences (Approval ID: Keshuizhuan 08529).

At each sampling time (except 20 dph), water samples were also collected from the left, center, and right of each tank as three replicates within tanks. Then, 500 mL of each water sample was sequentially filtered through 1.2-mm (Whatman, NJ, USA) and 0.22-mm filters (Millipore, MA USA) to collect microbial cells for evaluating the water microbiota^60^. We totally obtained 189 zebrafish gut samples (that is, 7 time points × 3 tanks × 3 cages × 3 replicates) and 54 water samples (that is, 6 time points × 3 tanks × 3 replicates) for following microbial analyses. The intestines and filters were immediately stored at −80 °C until DNA extraction. Microbial community DNA was extracted using the PowerFecal^®^ (gut samples) or PowerWater^®^ (water samples) DNA Isolation Kit (Mo Bio, CA, USA) following the manufacturer’s instructions. The concentration and quality of extracted DNA were determined using a NanoDrop One spectrophotometer (Thermo Fisher Scientific, MA, USA), and all the DNA samples were diluted to the same concentration (10 ng/μL) for subsequent PCR amplification.

### 16S rRNA gene amplicon sequencing and data analysis

The V4-V5 regions of the 16S rRNA gene were amplified by using the primer set of 515 F (5′-GTGCCAGCMGCCGCGGTAA-3′) and 907R (5′-CCGTCAATTCMTTTRAGTTT-3′). Each sample was amplified in a reaction volume of 50 μL containing 1× Premix Taq DNA polymerase (dNTP, Taq and buffer were included), 0.2 mM of each primer, and 50 ng genomic DNA. The program for PCR amplification included DNA pre-denaturation for 5 min at 95 °C, then 30 cycles of 30 s at 95 °C, 30 s at 52 °C, and 30 s at 72 °C, followed by a final extension at 72 °C for 10 min. Negative controls were always performed to make sure there is no contamination. After all samples were successfully amplified, the PCR products were quantified and equally combined. The target band visualized by 2.0% agarose gel was excised and purified with a QIAquick Gel Extraction Kit (Qiagen, CA, USA). After re-quantifying the concentration of purified DNA, it was subjected to library construction. The constructed amplicon library was finally sequenced by the Illumina HiSeq 2500 platform (Illumina, CA, USA) in Guangdong Magigene Biotechnology Co., Ltd. with a 2 × 250 bp kit.

Quality filtering and processing of sequence reads were conducted on the publicly available Galaxy pipeline (http://mem.rcees.ac.cn:8080/) as described previously^61^. In brief, the overlapped paired-end sequences were first assembled using QIIME (Quantitative Insights into Microbial Ecology)^62^, and poorly overlapped and low-quality sequences such as those with length <140 and moving-window (5 bp) quality score <20 were removed before downstream analysis. The table of zOTUs (starting now, called OTU) generated by the UNOISE method according to the database of Greengenes 13_8^63^. The singletons were removed from the original OTU table. All samples were resampled to a same sequencing depth (i.e., 14,666 sequences per sample) before subsequent community analysis and statistics.

### Ecological process analysis

The role of water and gut habitats for shaping the metacommunity in each tank or across all tanks was separated into their contributions at smaller scales from habitats to local communities as described previously^25^. In order to directly visualize the role of each habitat for shaping the metacommunity composition, we used an additive diversity partitioning framework to decompose the total diversity and expressed it as the sum of the diversity observable at various scales with *Rao* function^25^. Specifically, the overall diversity of ecosystem (γ_Ecosystem_) was partitioned into the sum of inter-habitat compositional differences (*β*_Inter-Habitats_; i.e., between water and gut habitats), the mean intra-habitat compositional differences (*β*_Intra-Habitats_; i.e., within water or gut habitats) and the mean diversity of local communities (*α*_Local-Communities_; i.e., each water or gut sample).

To quantify the influence of ecological processes on fish-associated microbial communities, the relative contribution of key processes (e.g., selection and dispersal) governing the gut microbiota succession was determined as described previously^16^. First, the representative sequences of each OTU were used to align with the 16 S GreenGene sequences using PyNAST^64^, and a maximum-likelihood tree was constructed using FastTree^65^. Then, phylogenetic diversity (PD) of each given pair of communities was quantified by the weighted beta nearest taxon index (βNTI)^66^. The βNTI in a combination of Bray-Curtis-based Raup-Crick (*RC*_bray_)^67,68^ was further used to quantify the ecological processes. In brief, the relative impact of community turnover determined by the heterogeneous selection and homogeneous selection can be indicated by the fraction of communities with βNTI > 2 and βNTI < −2, respectively^69^. These selection processes include environmental filtering, biotic interactions, nonrandom dispersal, and positive mutations^70^. If |βNTI| < 2, but with *RC*_bray_ > 0.95 or <−0.95 suggested that the community turnover is governed by dispersal limitation or homogenizing dispersal, respectively. However, if |βNTI| < 2 and |*RC*_bray_| < 0.95, that means the community turnover is not governed by any process as mentioned above and therefore classified as undominated process, which includes some weak selection, weak dispersal, diversification, and drift^70^.

### Statistical analysis

Multivariate regression tree (MRT) analysis^71^ was used to explore and predict relationships between the gut microbiota and expected influence factors (i.e., developmental stage, environment, transition, and food). This method has been widely used for modeling species-environment relationships, and it is well suited for complex ecological datasets with high-order interactions. General linear model (GLM) analysis was used to ascertain what underlying factors shaped the alpha-diversity (i.e., PD, richness, and Shannon). Hierarchical partitioning was performed to identify important potential causal variables with independent effects on the alpha-diversity. Mantel and partial Mantel tests were performed to determine relationships between environmental factors and the microbial community structure based on both Bray–Curtis and Jaccard distances.

In order to show a general pattern of the relatively dominant microbial members, the top OTUs in the gut or water were estimated by proportions based on averages per sample across all environments (A, B, and C). More specifically, the generated OTU table was analyzed tank-by-tank or compared across all tanks using the following statistical methods: (i) alpha- and beta-diversity comparisons were conducted to reveal changes in gut microbiotas throughout host development, or between water microbiota and gut microbiota; (ii) detrended correspondence analysis (DCA) was conducted to illustrate overall similarities of microbial communities based on the Bray-Curtis distances; (iii) permutational multivariate analysis of variance (PERMANOVA) was performed to evaluate the significance of community dissimilarities based on both Bray-Curtis and Jaccard distances^72^; (iv) in order to determine whether the observed community similarities within each stage are indistinguishable from the null expectation, the null model analysis based on the method proposed by Chase et al.^47^ by holding α-diversity and γ-diversity across total dataset constant. We performed this analysis based on the Bray–Curtis distance without data transformation. It provides a quantitative estimation of the role of deterministic selection processes in shaping community composition and structure, such ratio is termed as selection strength. (v) canonical correspondence analysis (CCA) was used to explain the contributions of the top 20 dominant OTUs to the overall compositional variation of the gut microbiota;^73^ vi) significance tests were performed through an analysis of variance (ANOVA) with least-significant-difference (LSD) to examine whether differences among comparisons were significant or not. All statistical analyses were performed using the R software (R Foundation for Statistical Computing, Vienna, Austria).

### Reporting summary

Further information on experimental design is available in the Nature Research Reporting Summary linked to this paper.

## Supplementary information

Supplementary information

Reporting Summary

## Data Availability

The raw sequencing data can be found at the National Centre for Biotechnology Information (NCBI) Sequence Read Archive (SRA) with an accession number PRJNA565801.
