# Supplementary material for: Host development overwhelms environmental dispersal in governing the ecological succession of zebrafish gut microbiota
Source: NPJ Biofilms Microbiomes. 2021 Jan 19;7:5. doi: 10.1038/s41522-020-00176-2 (PMC7815754; doi:10.1038/s41522-020-00176-2)
Supplement: Supplementary file 1 — Supplementary information [file 41522_2020_176_MOESM1_ESM.pdf]

Supplementary information

Host development overwhelms environmental dispersal in governing the ecological succession of zebrafish gut microbiota

Fanshu Xiao[1,9], Wengen Zhu[2,9], Yuhe Yu[2], Zhili He[1,3], Bo Wu[1], Cheng Wang[1], Longfei Shu[1], Xinghao Li[2], Huaqun Yin[4], Jianjun Wang[5], Philippe Juneau[6], Xiafei Zheng[1], Yongjie Wu[1], Juan Li[3], Xiaojuan Chen[7], Dongwei Hou[1], Zhijian Huang[1], Jianguo He[1], Guohuan Xu[8], Liwei Xie[8], Jie Huang[2]✉ and Qingyun Yan[1]✉

[1]Environmental Microbiomics Research Center, School of Environmental Science and Engineering, Southern Marine Science and Engineering Guangdong Laboratory (Zhuhai), Sun Yat-sen University, Guangzhou 510006, China. [2]Key Laboratory of Aquatic Biodiversity and Conservation of Chinese Academy of Sciences, Institute of Hydrobiology, Chinese Academy of Sciences, Wuhan 430072, China. [3]College of Agronomy, Hunan Agricultural University, Changsha 410128, China. [4]Key Laboratory of Biometallurgy of Ministry of Education, School of Minerals Processing and Bioengineering, Central South University, Changsha 410083, China. [5]State Key Laboratory of Lake Science and Environment, Nanjing Institute of Geography and Limnology, Chinese Academy of Sciences, Nanjing 210008, China. [6]Department of Biological Science, GRIL, TOXEN, Ecotoxicology of Aquatic Microorganisms Laboratory, Université du Québec à Montréal, Succursale Centre-Ville, Montréal, Québec, Canada. [7]Key Laboratory of Ecological Impacts of Hydraulic-Projects and Restoration of Aquatic Ecosystem of Ministry of Water Resources, Institute of Hydroecology, Ministry of Water Resources and Chinese Academy of Sciences, Wuhan 430079, China. [8]State Key Laboratory of Applied Microbiology Southern China, Guangdong Institute of Microbiology, Guangdong Academy of Sciences, Guangzhou 510070, China. [9]These authors contributed equally: Fanshu Xiao, Wengen Zhu. ✉email: yanqy6@mail.sysu.edu.cn; jhuang@ihb.ac.cn

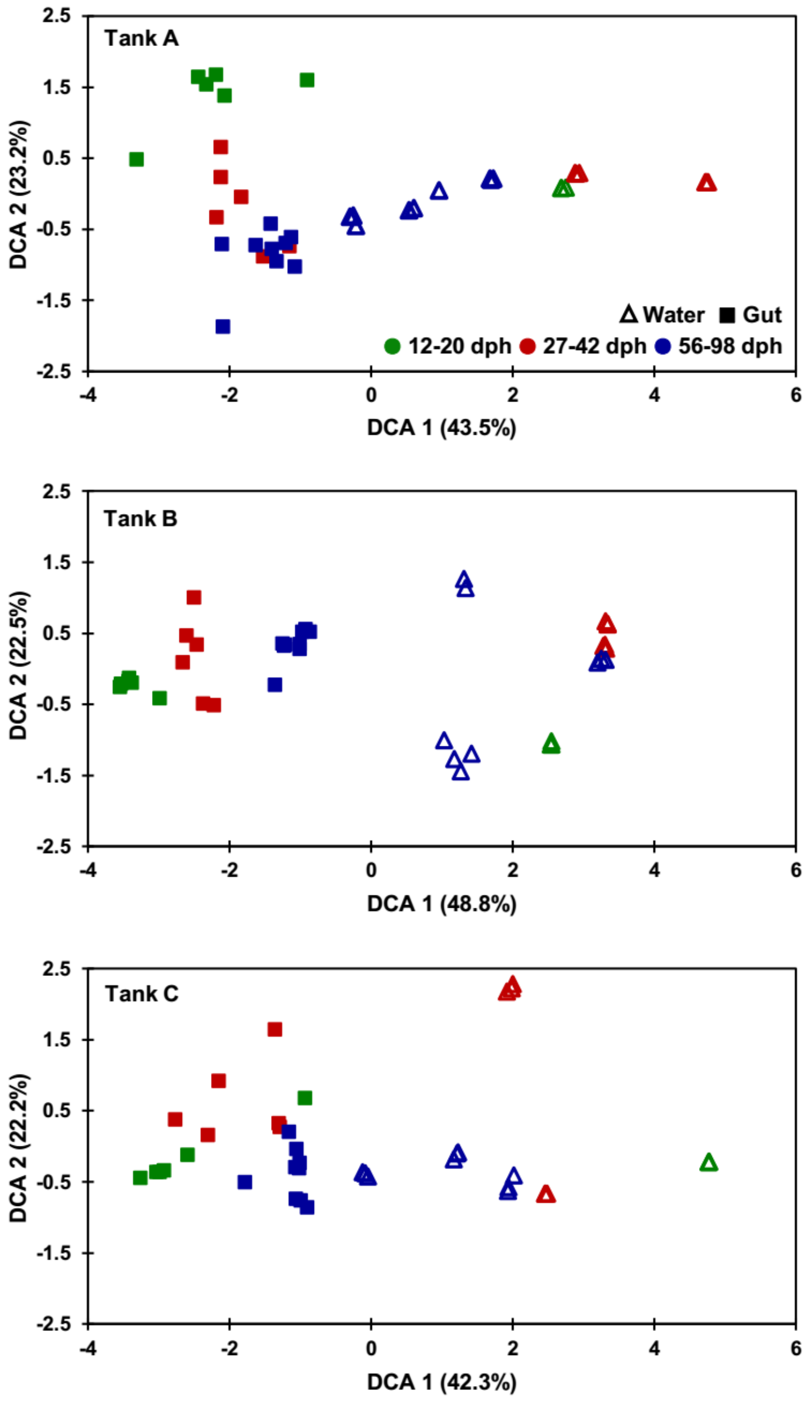

Supplementary Fig. 1 Detrended correspondence analysis (DCA) showing the dissimilarities of gut microbiotas and water microbiotas within each tank across zebrafish development. Zebrafish were raised in original environments. No water sample is available for 20-day post-hatching (dph).

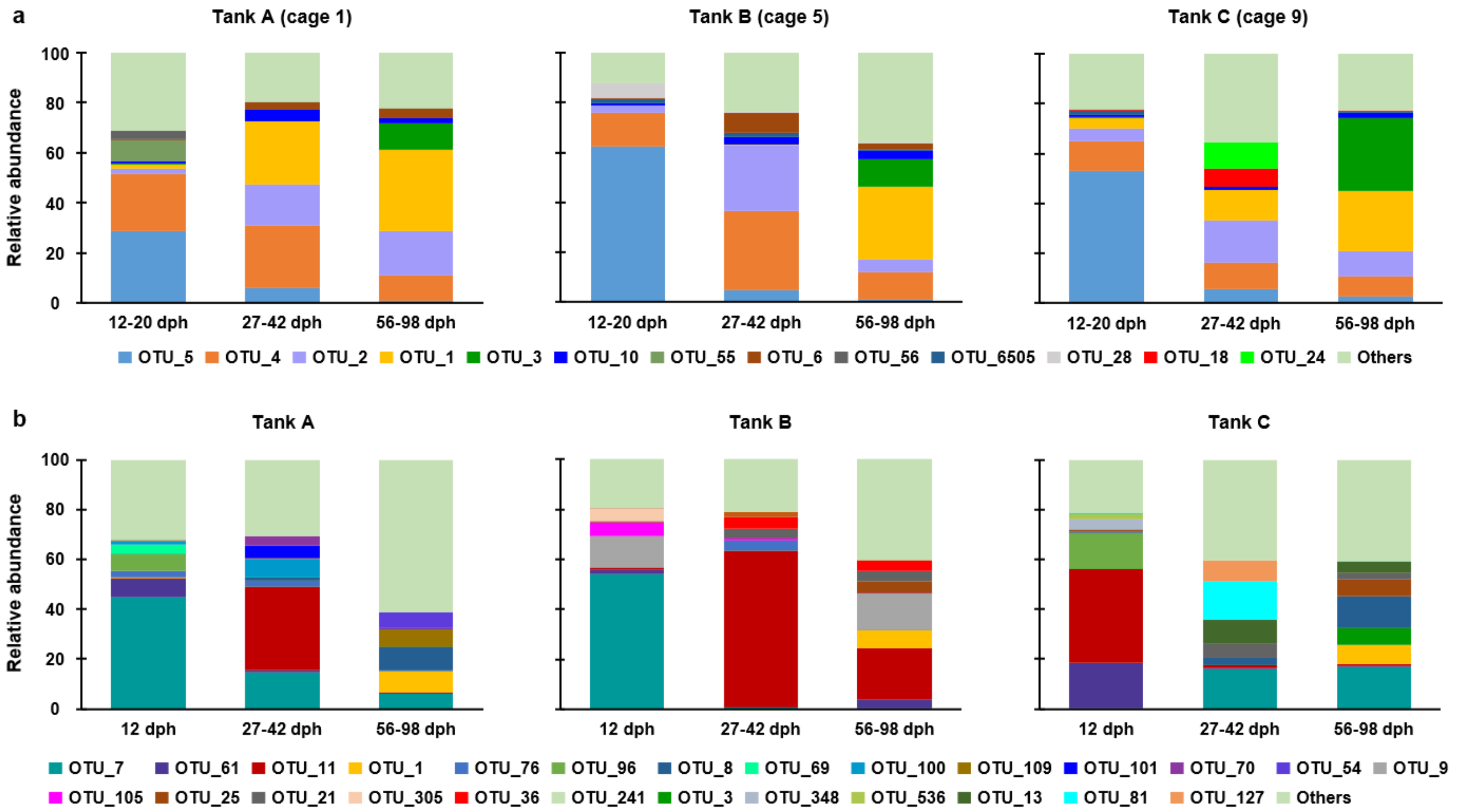

Supplementary Fig. 2 Comparison of relative abundances of the top 5 OTUs varies across zebrafish development within each tank. **a** gut microbiotas in zebrafish reared in original environments. In each tank, the top 5 OTUs detected in any stage were plotted throughout all stages to make sure the OTUs presented in different stages is consistent. Only the mean values of samples collected from each stage are plotted. dph: day post-hatching. **b** water microbiotas. OTU_1 and OTU_3: *Cetobacterium*; OTU_2: *Plesiomonas*; OTU_5 and OTU_6505: *Vibrio*; OTU_7, OTU_11 and OTU_81: *Flavobacterium*; OTU_8: *Clavibacter*; OTU_18: *Citrobacter*; OTU_21, OTU_109, and OTU_70: *Limnohabitans*; OTU_25: *Mycobacterium*; OTU_28: *Shewanella*; OTU_36: *Sediminibacterium*; OTU_54: *Cloacibacterium*; OTU_55: *Acinetobacter*; OTU_56: *Anaerospora*; OTU_61: *Rhodoferax*; OTU_76: *Acidovorax*; OTU_96: *Armatimonas*; OTU_105: *Polynucleobacter*; OTU_241: *Candidatus* Rhodoluna; OTU_536: *Phenylobacterium*; other OTUs: unclassified at the genus level.

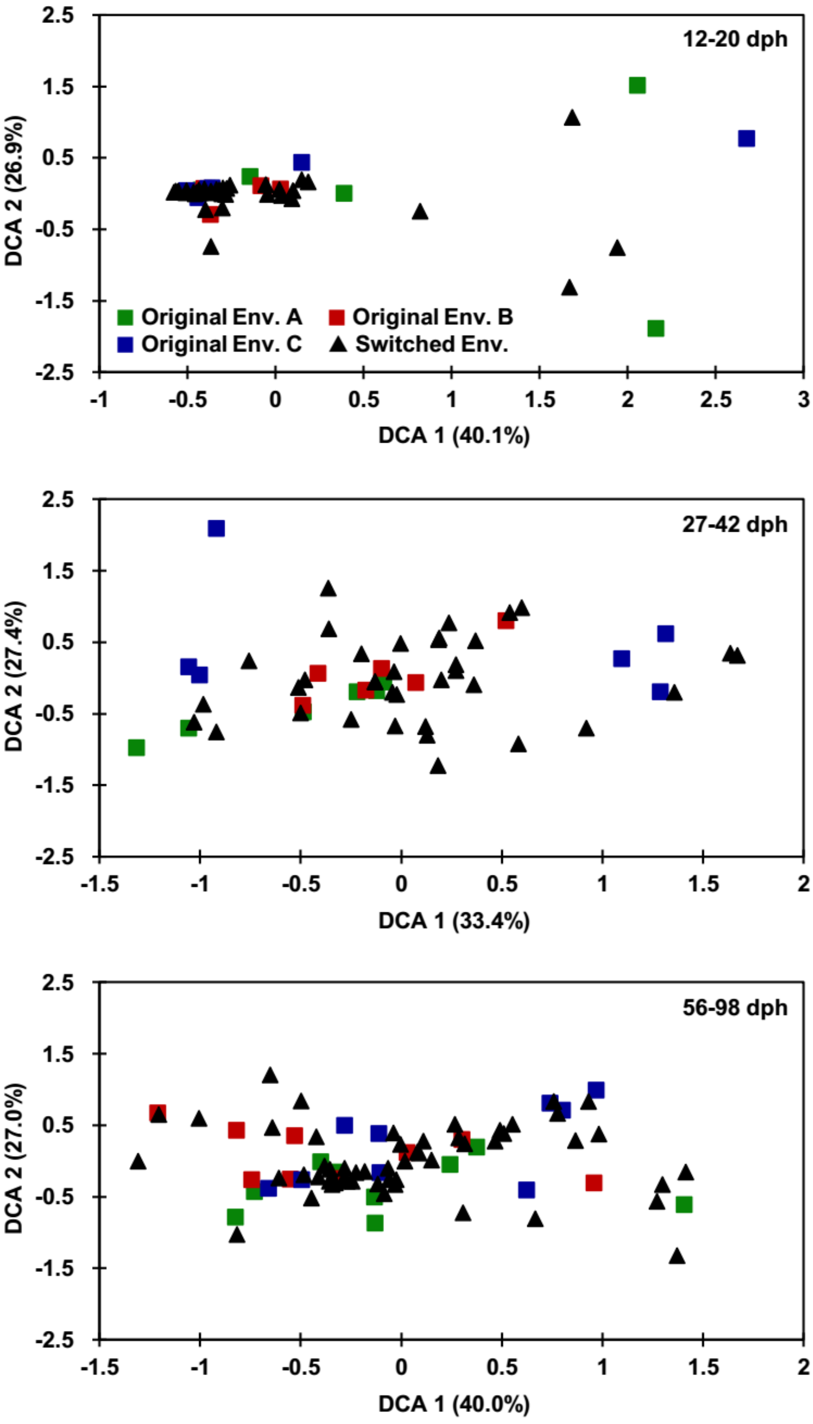

Supplementary Fig. 3 Detrended correspondence analysis (DCA) showing the dissimilarities of gut microbiota of zebrafish raised in original environments (Env.) and switched environments according to developmental stages. dph: day post-hatching.

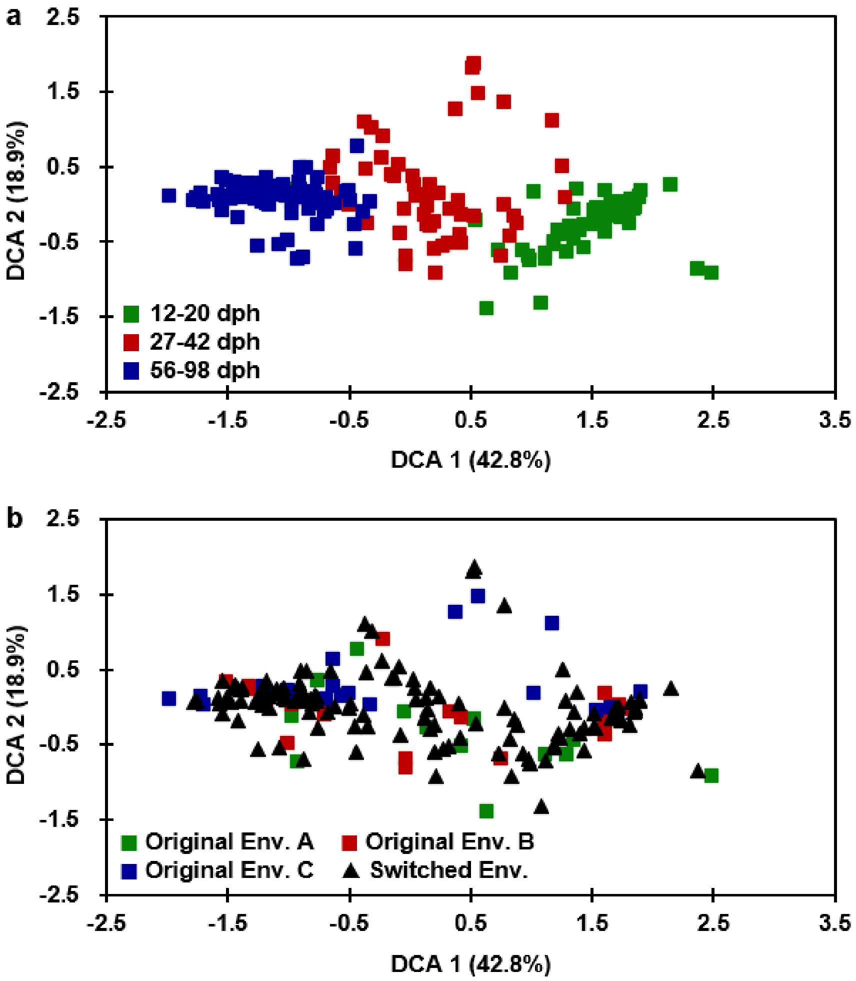

Supplementary Fig. 4 Detrended correspondence analysis (DCA) showing the dissimilarities of gut microbiota in zebrafish. **a** according to developmental stages. **b** according to environments (Env.). dph: day post-hatching.

Supplementary Table 1. Taxonomy and classification of OTUs showed in Fig. 3 or Supplementary Fig. 2.

OTU ID	Phylum	Class	Order	Family	Genus
OTU_1	Fusobacteria	Fusobacteriia	Fusobacteriales	Fusobacteriaceae	*Cetobacterium*
OTU_2	Proteobacteria	Gammaproteobacteria	Enterobacteriales	Enterobacteriaceae	*Plesiomonas*
OTU_3	Fusobacteria	Fusobacteriia	Fusobacteriales	Fusobacteriaceae	*Cetobacterium*
OTU_4	Proteobacteria	Gammaproteobacteria	Aeromonadales	Aeromonadaceae	Unclassified
OTU_5	Proteobacteria	Gammaproteobacteria	Vibrionales	Pseudoalteromonadaceae	*Vibrio*
OTU_6	Proteobacteria	Gammaproteobacteria	Vibrionales	Pseudoalteromonadaceae	Unclassified
OTU_7	Bacteroidetes	Flavobacteriia	Flavobacteriales	Flavobacteriaceae	*Flavobacterium*
OTU_8	Actinobacteria	Actinobacteria	Actinomycetales	Microbacteriaceae	*Clavibacter*
OTU_9	Proteobacteria	Alphaproteobacteria	Rhizobiales	Unclassified	Unclassified
OTU_10	Proteobacteria	Gammaproteobacteria	Aeromonadales	Aeromonadaceae	Unclassified
OTU_11	Bacteroidetes	Flavobacteriia	Flavobacteriales	Flavobacteriaceae	*Flavobacterium*
OTU_13	Actinobacteria	Actinobacteria	Actinomycetales	ACK-M1	Unclassified
OTU_15	Proteobacteria	Gammaproteobacteria	Vibrionales	Vibrionaceae	*Vibrio*
OTU_16	Bacteroidetes	Saprospirae	Saprospirales	Chitinophagaceae	*Flavihumibacter*
OTU_18	Proteobacteria	Gammaproteobacteria	Enterobacteriales	Enterobacteriaceae	*Citrobacter*
OTU_19	Proteobacteria	Betaproteobacteria	Burkholderiales	Oxalobacteraceae	*Polynucleobacter*
OTU_20	TM6	SJA-4	Unclassified	Unclassified	Unclassified
OTU_21	Proteobacteria	Betaproteobacteria	Burkholderiales	Comamonadaceae	*Limnohabitans*
OTU_22	Firmicutes	Clostridia	Clostridiales	Peptostreptococcaceae	Unclassified
OTU_23	Proteobacteria	Gammaproteobacteria	Alteromonadales	Shewanellaceae	*Shewanella*
OTU_24	Proteobacteria	Alphaproteobacteria	Rickettsiales	mitochondria	Unclassified
OTU_25	Actinobacteria	Actinobacteria	Actinomycetales	Mycobacteriaceae	*Mycobacterium*
OTU_26	Proteobacteria	Gammaproteobacteria	Vibrionales	Pseudoalteromonadaceae	Unclassified
OTU_27	Proteobacteria	Gammaproteobacteria	Vibrionales	Pseudoalteromonadaceae	Unclassified
OTU_28	Proteobacteria	Gammaproteobacteria	Alteromonadales	Shewanellaceae	*Shewanella*

OTU ID	Phylum	Class	Order	Family	Genus
OTU_29	Tenericutes	CK-1C4-19	Unclassified	Unclassified	Unclassified
OTU_30	Proteobacteria	Gammaproteobacteria	Alteromonadales	Shewanellaceae	*Shewanella*
OTU_32	Proteobacteria	Gammaproteobacteria	Xanthomonadales	Xanthomonadaceae	Unclassified
OTU_36	Bacteroidetes	Saprospirae	Saprospirales	Chitinophagaceae	*Sediminibacterium*
OTU_41	Proteobacteria	Alphaproteobacteria	Rhodobacterales	Rhodobacteraceae	Unclassified
OTU_42	Proteobacteria	Gammaproteobacteria	Pseudomonadales	Pseudomonadaceae	*Pseudomonas*
OTU_53	Proteobacteria	Gammaproteobacteria	Alteromonadales	OM60	Unclassified
OTU_54	Bacteroidetes	Flavobacteriia	Flavobacteriales	Weeksellaceae	*Cloacibacterium*
OTU_55	Proteobacteria	Gammaproteobacteria	Pseudomonadales	Moraxellaceae	*Acinetobacter*
OTU_56	Proteobacteria	Alphaproteobacteria	Rhodobacterales	Rhodobacteraceae	*Anaerospora*
OTU_61	Proteobacteria	Betaproteobacteria	Burkholderiales	Comamonadaceae	*Rhodoferax*
OTU_69	Actinobacteria	Actinobacteria	Actinomycetales	ACK-M1	Unclassified
OTU_70	Proteobacteria	Betaproteobacteria	Burkholderiales	Comamonadaceae	*Limnohabitans*
OTU_76	Proteobacteria	Betaproteobacteria	Burkholderiales	Comamonadaceae	*Acidovorax*
OTU_77	Proteobacteria	Gammaproteobacteria	Enterobacteriales	Enterobacteriaceae	Unclassified
OTU_78	Proteobacteria	Alphaproteobacteria	Rickettsiales	Unclassified	Unclassified
OTU_79	Bacteroidetes	Flavobacteriia	Flavobacteriales	Weeksellaceae	*Wautersiella*
OTU_80	Proteobacteria	Gammaproteobacteria	Enterobacteriales	Enterobacteriaceae	*Klebsiella*
OTU_81	Bacteroidetes	Flavobacteriia	Flavobacteriales	Flavobacteriaceae	*Flavobacterium*
OTU_85	Proteobacteria	Betaproteobacteria	Burkholderiales	Comamonadaceae	Unclassified
OTU_91	Bacteroidetes	Saprospirae	Saprospirales	Chitinophagaceae	*Sediminibacterium*
OTU_96	Armatimonadetes	Armatimonadia	Armatimonadales	Armatimonadaceae	*Armatimonas*
OTU_100	Bacteroidetes	Cytophagia	Cytophagales	Cytophagaceae	Unclassified
OTU_101	Bacteroidetes	Sphingobacteriia	Sphingobacteriales	Unclassified	Unclassified
OTU_105	Proteobacteria	Betaproteobacteria	Burkholderiales	Oxalobacteraceae	*Polynucleobacter*
OTU_109	Proteobacteria	Betaproteobacteria	Burkholderiales	Comamonadaceae	*Limnohabitans*
OTU_117	Proteobacteria	Alphaproteobacteria	Rhizobiales	Rhizobiaceae	Unclassified

OTU ID	Phylum	Class	Order	Family	Genus
OTU_122	Spirochaetes	Leptospirae	Leptospirales	Leptospiraceae	*Leptospira*
OTU_123	Proteobacteria	Betaproteobacteria	Methylophilales	Methylophilaceae	Unclassified
OTU_127	Bacteroidetes	Sphingobacteriia	Sphingobacteriales	Sphingobacteriaceae	Unclassified
OTU_144	Proteobacteria	Betaproteobacteria	Burkholderiales	Comamonadaceae	*Acidovorax*
OTU_150	Planctomycetes	Planctomycetia	Pirellulales	Pirellulaceae	Unclassified
OTU_214	Proteobacteria	Alphaproteobacteria	Rhizobiales	Unclassified	Unclassified
OTU_223	Proteobacteria	Alphaproteobacteria	Sphingomonadales	Sphingomonadaceae	*Novosphingobium*
OTU_241	Actinobacteria	Actinobacteria	Actinomycetales	Microbacteriaceae	*Candidatus* Rhodoluna
OTU_288	Proteobacteria	Alphaproteobacteria	Sphingomonadales	Sphingomonadaceae	*Novosphingobium*
OTU_305	Verrucomicrobia	Pedosphaerae	Pedosphaerales	R4-41B	Unclassified
OTU_329	Proteobacteria	Betaproteobacteria	Burkholderiales	Comamonadaceae	Unclassified
OTU_348	Actinobacteria	Actinobacteria	Actinomycetales	Unclassified	Unclassified
OTU_384	Actinobacteria	Actinobacteria	Actinomycetales	ACK-M1	Unclassified
OTU_396	Bacteroidetes	Flavobacteriia	Flavobacteriales	Cryomorphaceae	*Fluviicola*
OTU_416	Proteobacteria	Betaproteobacteria	Methylophilales	Methylophilaceae	Unclassified
OTU_429	Actinobacteria	Actinobacteria	Actinomycetales	ACK-M1	Unclassified
OTU_433	Proteobacteria	Betaproteobacteria	Burkholderiales	Comamonadaceae	*Limnohabitans*
OTU_460	Actinobacteria	Actinobacteria	Actinomycetales	Mycobacteriaceae	*Mycobacterium*
OTU_469	Bacteroidetes	Cytophagia	Cytophagales	Cytophagaceae	Unclassified
OTU_471	Proteobacteria	Betaproteobacteria	Burkholderiales	Comamonadaceae	*Acidovorax*
OTU_503	Bacteroidetes	Flavobacteriia	Flavobacteriales	Flavobacteriaceae	*Flavobacterium*
OTU_536	Proteobacteria	Alphaproteobacteria	Caulobacterales	Caulobacteraceae	*Phenylobacterium*
OTU_595	Proteobacteria	Alphaproteobacteria	Rickettsiales	Rickettsiaceae	*Rickettsia*
OTU_611	Proteobacteria	Betaproteobacteria	Burkholderiales	Unclassified	Unclassified
OTU_671	Proteobacteria	Betaproteobacteria	Methylophilales	Methylophilaceae	Unclassified
OTU_703	Proteobacteria	Alphaproteobacteria	Rhizobiales	Unclassified	Unclassified
OTU_710	Bacteroidetes	Flavobacteriia	Flavobacteriales	Flavobacteriaceae	*Flavobacterium*

OTU ID	Phylum	Class	Order	Family	Genus
OTU_719	Actinobacteria	Actinobacteria	Actinomycetales	ACK-M1	Unclassified
OTU_760	Bacteroidetes	Flavobacteriia	Flavobacteriales	Flavobacteriaceae	*Flavobacterium*
OTU_766	Proteobacteria	Betaproteobacteria	Methylophilales	Methylophilaceae	Unclassified
OTU_786	Bacteroidetes	Saprospirae	Saprospirales	Chitinophagaceae	*Sediminibacterium*
OTU_793	Proteobacteria	Betaproteobacteria	Methylophilales	Methylophilaceae	Unclassified
OTU_807	Proteobacteria	Betaproteobacteria	Burkholderiales	Oxalobacteraceae	Unclassified
OTU_881	Proteobacteria	Betaproteobacteria	Burkholderiales	Comamonadaceae	*Diaphorobacter*
OTU_980	Bacteroidetes	Sphingobacteriia	Sphingobacteriales	Unclassified	Unclassified
OTU_1011	Planctomycetes	Planctomycetia	Pirellulales	Pirellulaceae	Unclassified
OTU_1122	Actinobacteria	Thermoleophilia	Gaiellales	Unclassified	Unclassified
OTU_1283	Bacteroidetes	Sphingobacteriia	Sphingobacteriales	Unclassified	Unclassified
OTU_1297	Proteobacteria	Betaproteobacteria	Burkholderiales	Unclassified	Unclassified
OTU_1350	Actinobacteria	Actinobacteria	Actinomycetales	Unclassified	Unclassified
OTU_1367	Bacteroidetes	Bacteroidia	Bacteroidales	Unclassified	Unclassified
OTU_1426	Proteobacteria	Betaproteobacteria	Burkholderiales	Comamonadaceae	*Rubrivivax*
OTU_1527	OD1	ZB2	Unclassified	Unclassified	Unclassified
OTU_1598	OD1	ZB2	Unclassified	Unclassified	Unclassified
OTU_1909	Acidobacteria	Solibacteres	Solibacterales	Unclassified	Unclassified
OTU_1959	Proteobacteria	Betaproteobacteria	Burkholderiales	Comamonadaceae	Unclassified
OTU_1970	Proteobacteria	Alphaproteobacteria	Rhizobiales	Unclassified	Unclassified
OTU_2071	Bacteroidetes	Flavobacteriia	Flavobacteriales	Cryomorphaceae	*Fluviicola*
OTU_2408	Proteobacteria	Betaproteobacteria	Burkholderiales	Oxalobacteraceae	Unclassified
OTU_2443	Actinobacteria	Actinobacteria	Actinomycetales	ACK-M1	Unclassified
OTU_3834	Proteobacteria	Betaproteobacteria	Burkholderiales	Oxalobacteraceae	*Polynucleobacter*
OTU_5691	Proteobacteria	Betaproteobacteria	Burkholderiales	Oxalobacteraceae	*Polynucleobacter*
OTU_6505	Proteobacteria	Gammaproteobacteria	Vibrionales	Pseudoalteromonadaceae	*Vibrio*
OTU_6704	Actinobacteria	Actinobacteria	Actinomycetales	ACK-M1	Unclassified

Supplementary Table 2. Permutational multivariate analysis of variance (PERMANOVA) showing the gut microbiota differences between zebrafish raised in original environments (Env.) and switched environments.

	Bray-Curtis	
	F	*p*
12-20 dph		
Original Env. A vs Switched Env.	1.48	0.180
Original Env. B vs Switched Env.	1.45	0.209
Original Env. C vs Switched Env.	1.38	0.199
27-42 dph		
Original Env. A vs Switched Env.	1.45	0.186
Original Env. B vs Switched Env.	0.80	0.592
Original Env. C vs Env. changed	2.63	0.014
56-98 dph		
Original Env. A vs Env. changed	1.13	0.282
Original Env. B vs Env. changed	2.06	0.068
Original Env. C vs Env. changed	2.03	0.071
across all stages		
Original Env. A vs Original Env. B	1.08	0.330
Original Env. A vs Original Env. C	1.56	0.143
Original Env. B vs Original Env. C	1.32	0.239
Original Env. A vs Switched Env.	0.65	0.640
Original Env. B vs Switched Env.	0.65	0.666
Original Env. C vs Switched Env.	1.41	0.197

dph: day post-hatching.

Supplementary Table 3. Permutational multivariate analysis of variance (PERMANOVA) showing the community differences between gut microbiotas and water microbiotas.

	Bray-Curtis		Jaccard	
	F	*p*	*F*	*p*
Hatched in environment A but raised in different tanks				
Gut 12-20 dph vs Water 12 dph	14.64	0.001	3.96	0.002
Gut 27-42 dph vs Water 27-42 dph	15.38	0.001	7.07	0.001
Gut 56-98 dph vs Water 56-98 dph	15.29	0.001	5.17	0.001
Hatched in environment B but raised in different tanks				
Gut 12-20 dph vs Water 12 dph	13.40	0.001	4.53	0.001
Gut 27-42 dph vs Water 27-42 dph	18.21	0.001	6.17	0.001
Gut 56-98 dph vs Water 56-98 dph	19.66	0.001	5.06	0.001
Hatched in environment C but raised in different tanks				
Gut 12-20 dph vs Water 12 dph	19.07	0.001	3.79	0.003
Gut 27-42 dph vs Water 27-42 dph	12.81	0.001	5.86	0.001
Gut 56-98 dph vs Water 56-98 dph	14.80	0.001	4.47	0.001
Hatched from different environments but raised in tank A				
Gut 12-20 dph vs Water 12 dph	10.10	0.001	3.85	0.001
Gut 27-42 dph vs Water 27-42 dph	17.46	0.001	7.21	0.001
Gut 56-98 dph vs Water 56-98 dph	17.87	0.001	5.57	0.001
Hatched from different environments but raised in tank B				
Gut 12-20 dph vs Water 12 dph	37.39	0.001	4.96	0.002
Gut 27-42 dph vs Water 27-42 dph	34.44	0.001	7.53	0.001
Gut 56-98 dph vs Water 56-98 dph	18.99	0.001	6.58	0.001
Hatched from different environments but raised in tank C				
Gut 12-20 dph vs Water 12 dph	15.11	0.001	4.43	0.001
Gut 27-42 dph vs Water 27-42 dph	10.37	0.001	5.68	0.001
Gut 56-98 dph vs Water 56-98 dph	16.29	0.001	5.25	0.001
Across all tanks				
Gut 12-20 dph vs Water 12 dph	31.66	0.001	8.17	0.001
Gut 27-42 dph vs Water 27-42 dph	33.62	0.001	14.93	0.001
Gut 56-98 dph vs Water 56-98 dph	41.72	0.001	11.73	0.001

No water sample is available for 20 days post-hatching (dph).

Supplementary Table 4. Summary statistics of general linear model (GLM) analysis of alpha diversity versus developmental stage, environment, transition and food.

Source	df	Phylogenetic diversity (PD)			
		Adj SS	Adj MS	*F*	*p*
Developmental stage	6	6817.00	1136.12	25.75	**< 0.001**
			r^2 = 45.91%		r^2(adj) = 44.13%
Environment	2	204.50	102.25	1.30	0.275
			r^2 = 1.38%		r^2(adj) = 0.32%
Transition	1	160.20	160.24	2.04	0.155
			r^2 = 1.08%		r^2(adj) = 0.55%
Food	1	124.60	124.57	1.58	0.210
			r^2 = 0.84%		r^2(adj) = 0.31%
		Richness			
		Adj SS	Adj MS	*F*	*p*
Developmental stage	6	4544157	757360	21.42	**< 0.001**
			r^2 = 41.39%		r^2(adj) = 39.45%
Environment	2	369338	184669	3.24	**0.041**
			r^2 = 3.36%		r^2(adj) = 2.32%
Transition	1	117943	117943	2.03	0.156
			r^2 = 1.07%		r^2(adj) = 0.55%
Food	1	85228	85228	1.42	0.228
			r^2 = 0.78%		r^2(adj) = 0.25%
		Shannon			
		Adj SS	Adj MS	*F*	*p*
Developmental stage	6	39.93	6.66	15.23	**< 0.001**
			r^2 = 33.43%		r^2(adj) = 31.24%
Environment	2	3.62	1.81	2.91	0.057
			r^2 = 3.03%		r^2(adj) = 1.99%
Transition	1	1.69	1.69	2.69	0.103
			r^2 = 1.42%		r^2(adj) = 0.89%
Food	1	0.08	0.08	0.12	0.727
			r^2 = 0.07%		r^2(adj) = 0.00%

The *p* values < 0.05 in bold.

Supplementary Table 5. Hierarchical partitioning of the variance in the alpha diversity associated with four predictors.

	Developmental stage	Environment	Transition	Food
Phylogenetic diversity (PD)				
Independent	0.152	0.002	0.011	0.020
Joint	-0.011	2.1E-17	-5.2E-18	-0.011
	**82.4%**	**1.2%**	**5.8%**	**10.6%**
Richness				
Independent	0.117	0.007	0.011	0.015
Joint	-0.007	-2.0E-17	1.6E-17	-0.007
	**78.5%**	**4.5%**	**7.2%**	**9.7%**
Shannon				
Independent	0.062	0.009	0.014	0.011
Joint	-0.010	-2.1E-17	-1.0E-17	-0.010
	**64.3%**	**9.8%**	**14.8%**	**11.1%**

The negative values for joint effects indicate that a variable is acting as a suppressor variable.

Supplementary Table 6. Summary statistics for Mantel and partial Mantel tests of correlation between gut microbiota (*M*) and environment (*E*) / transition (*T*) / food (*F*).

Test type	Test statistic	Bray-Curtis		Jaccard	
		r	*p*	*r*	*p*
Mantel	r(*ME*)	0.02	0.014	0.12	< 0.001
	r(*MT*)	-0.01	0.173	0.01	0.417
	r(*MF*)	0.21	< 0.001	0.10	< 0.001
partial Mantel	r(*ME.S*)	0.04	< 0.001	0.16	< 0.001
	r(*ME.T*)	0.02	0.031	0.11	< 0.001
	r(*ME.F*)	0.02	0.008	0.11	< 0.001
	r(*MT.S*)	0.01	0.534	0.01	0.116
	r(*MT.E*)	-0.01	0.061	-0.01	0.246
	r(*MT.F*)	-0.01	0.067	-0.003	0.708
	r(*MF.S*)	-0.14	< 0.001	-0.16	< 0.001
	r(*MF.E*)	0.16	< 0.001	0.07	< 0.001
	r(*MF.F*)	0.17	< 0.001	0.08	< 0.001

The Mantel statistic *r*(*AB*) estimates the correlation between two matrices *A* and *B*. Whereas, the partial Mantel *r*(*AB.C*) statistic estimates the correlation between *A* and *B* whilst controlling for the effects of *C*. The *S* indicates developmental stage.

Supplementary Table 7. Bray-Curtis distance-based significance tests of centroid differences between the observed communities and the null model simulations within zebrafish developmental stages.

	Observed similarity	Permutated similarity	Effect size	Deterministic ratio	*p*
Tank A					
12-20 dph	0.397	0.024	2.583	0.887	< 0.001
27-42 dph	0.483	0.028	2.843	0.933	< 0.001
56-98 dph	0.560	0.022	3.355	0.960	< 0.001
Tank B					
12-20 dph	0.655	0.017	3.656	0.972	< 0.001
27-42 dph	0.513	0.025	2.990	0.946	< 0.001
56-98 dph	0.531	0.036	2.733	0.927	< 0.001
Tank C					
12-20 dph	0.506	0.015	3.356	0.951	< 0.001
27-42 dph	0.316	0.024	2.533	0.907	< 0.001
56-98 dph	0.535	0.023	3.175	0.953	< 0.001
Three tanks overall					
12-20 dph	0.507	0.017	3.248	0.941	< 0.001
27-42 dph	0.394	0.024	2.767	0.927	< 0.001
56-98 dph	0.517	0.023	3.192	0.952	< 0.001

The *p* values are from the *t*-test on observed and permutated similarity. dph: day post-hatching.
